# Transforming Global Neurosurgical Education Through Mixed Reality Development: A 12-Year Bibliometric Analysis

**DOI:** 10.7759/cureus.95694

**Published:** 2025-10-29

**Authors:** Juan P Giraldo, Steve S Cho, Nikhil Dholaria, Brayden Haberman, Chinami Michaels, S. Harrison Farber, Juan S Uribe

**Affiliations:** 1 Department of Neurosurgery, Barrow Neurological Institute, St. Joseph's Hospital and Medical Center, Phoenix, USA

**Keywords:** bibliometric analysis, global, mixed reality, mixed-reality modules, neurosurgical education, neurosurgical procedures, technology

## Abstract

This study visualizes and analyzes global research collaborations, their current state, and the developmental trends of mixed reality (MR) education modules to predict future directions in neurosurgery. A structured review was conducted, focusing on MR, education, and neurosurgery, using Medline, Scopus, and Embase databases from January 1, 2013, to January 31, 2024. Search terms included (“mixed reality”) AND (education) AND (neurosurgery). Data on application purposes, design, and geographic locations were extracted. The global distribution and collaboration in emerging neurosurgical MR technologies were assessed using qualitative data extraction and bibliometric analyses. The search yielded 1,063 articles; 127 duplicates were removed. The remaining 936 articles were screened for relevance by two independent authors. Twenty-five articles on MR development or use, authored by 181 individuals from 21 countries, were included. Of these articles, 17 (68%) focused on cranial MR, two (8%) on spine MR, and six (24%) on both cranial and spine or unspecified neurosurgical MR training. Authors from China and the United States contributed the most articles (six each) and made significant contributions to the field, despite having limited international collaborations (one each). Both countries also had the most single-center publications (four each). The United States had the highest citation count (150). The leading journal publishing neurosurgical MR developments was *Neurosurgical Focus*, with six (24%) publications. The most common keywords were “mixed reality” (14), “neurosurgery” (five), and “augmented reality” (four), with 40, 20, and 13 connections to other keywords, respectively. MR applications in neurosurgery have a significant impact on trainee education. Current prototypes and applications complement traditional apprenticeship models, and it is anticipated that MR will increasingly integrate into neurosurgical curricula as more institutions allocate resources to develop this emerging technology through international collaboration. Additionally, MR applications offer a safe training environment.

## Introduction and background

Background of emerging technologies

Neurosurgical training and education have evolved in step with the pace of novel technological advancements. Virtual reality, augmented reality, and mixed reality (MR) complement multiple spheres of medicine, including the traditional apprenticeship models of medical training. In the case of neurosurgery training, emerging evidence suggests that these interreality systems provide educational settings that facilitate comprehension and skill mastery [1]. Among these tools, MR has evolved to encompass the spectrum of the virtual reality continuum, where its current definition describes the merging of a real-world environment and a computer-generated one [2,3]. MR refers to the blending of real and virtual environments, enabling real-time interaction between physical and digital objects. Unlike virtual reality, which immerses users entirely in a computer-generated environment, and augmented reality, which overlays digital elements onto the real world, MR allows for dynamic, spatially aware engagement with both realms simultaneously. Common MR devices include the Microsoft HoloLens (Microsoft Corporation, Washington, United States) and Magic Leap (Magic Leap, Inc., Florida, United States), which enable immersive surgical simulations and real-time anatomical guidance.

Mixed reality technology as an educational tool

Throughout the world, MR is being used in neurosurgical training centers for several procedural training courses, and the impact of this training is being shared in the literature, exposing a trend of MR incorporation into neurosurgical training, education, and patient care. This comprehensive, structured literature review explores the impact and prevalence of MR modules in neurosurgical education and training programs worldwide through bibliometric analyses to examine the history and research development of MR integration over a 12-year period. Bibliometric analysis was selected due to its ability to quantify research productivity, map collaboration networks, and identify keyword trends over time, which are metrics particularly suited to evaluating the evolution of a niche, yet rapidly expanding, educational tool. This study investigated the extent to which MR modeling is incorporated into global neurosurgical training, emphasizing collaborative efforts among institutions and countries to foresee the direction of new MR applications within neurosurgical education, with the aim of answering the following research question: How has MR been integrated into neurosurgical education globally over the past 12 years, and what are the trends and collaborative networks shaping its development?

## Review

Search strategy

**Figure 1:**
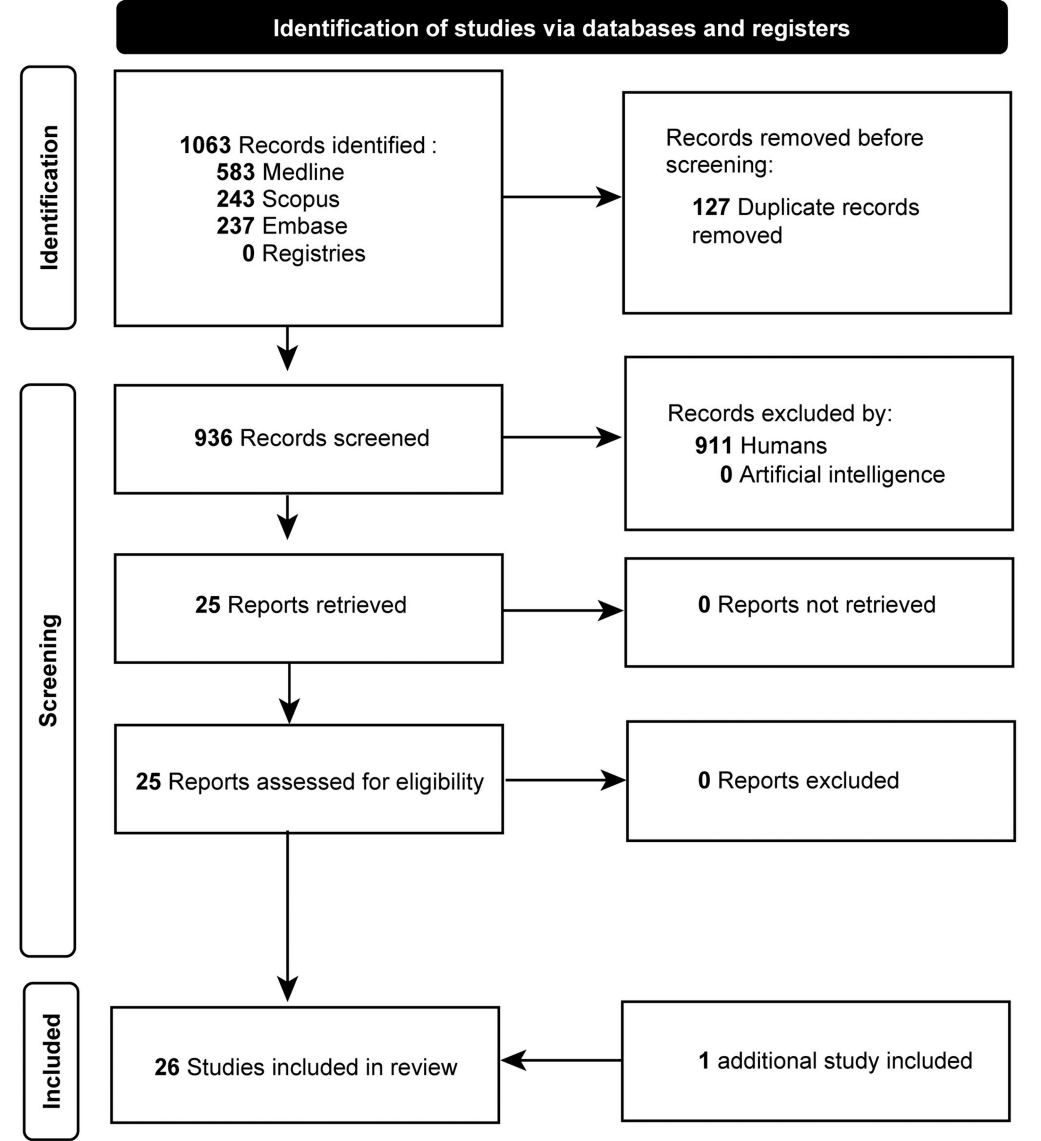
Search strategy flow diagram Used with permission from Barrow Neurological Institute, Phoenix, Arizona.

A comprehensive, structured literature search of MR, neurosurgery, and education within the previous 12 years was implemented using Medline, Scopus, and Embase databases. An evaluation of search engines and databases was performed to ensure that no other bibliometric analysis or systematic review was previously performed on this topic. An automated search strategy was conducted for the period from January 1, 2013, to January 31, 2024, using the following search terms: (“mixed reality”) AND (education) AND (neurosurgery). Two authors (N.D. and B.H.) independently screened the titles and abstracts of all publications for relevance, with full-text reviews of certain publications subjected to more detailed examinations. Discrepancies during the review process were resolved through mutual agreement with the senior author (J.S.U.). Institutional review board approval was not required for this systematic literature review. This review adheres to Preferred Reporting Items for Systematic Reviews and Meta-Analyses (PRISMA) guidelines in documenting the selection and synthesis of included studies (Figure 1).

Following revisions, an additional study from January 2025 was incorporated to extend the bibliometric analysis to the most current data available.

Inclusion and exclusion criteria

The inclusion criteria encompassed all publications relevant to an analysis of the geographic location of MR use in neurosurgery. Inclusion criteria were English-language articles and studies involving MR use in neurosurgery only, including cranial and spinal use. Papers that used MR for neurosurgical procedures and neurosurgical training were included. Exclusion criteria were publications that used MR modalities not specifically directed to the neurosurgical specialty, publications in languages other than English, and abstract-only publications. Gray literature, such as conference abstracts, technical reports, and non-peer-reviewed materials, was excluded to ensure methodological consistency and citation verifiability. Non-English language studies were excluded due to translation resource limitations. The search results were compared to the papers’ respective findings to ensure the integrity and accuracy of the search results. The detailed search strategy is shown in Figure 1. Studies were not excluded based on sample size alone; however, sample size was documented for each study and descriptively analyzed to assess study scope. No weighting adjustments were applied based on sample size. An additional study identified in January 2025 was included during revisions to ensure the bibliometric analysis reflected the most up-to-date literature.

Data extraction and analysis

The geographic locations and institutions for each article, including the individual locations of each author, were extracted from the included studies. Additionally, the type of study, the indication of MR use, the MR device, the number of participants, and the major findings of each study were analyzed. The bibliometric analysis was performed using VOSviewer (Centre for Science and Technology Studies, Leiden University, The Netherlands), a tool that visualizes bibliometric networks [4]. The tool groups closely related nodes into clusters and indicates higher levels of correlation with similar or identical colors among the nodes. The relative size of the node also represents the frequency of the specific variable of interest. Additionally, VOSviewer supports three visualization maps: network visualization, overlay visualization, and density visualization. In this study, VOSviewer was employed to visualize the network of collaboration between countries and describe the relationships between keywords in the included articles. Nodes with no connections were excluded from the VOSviewer visualization maps. A world map graphic was used to show the MR module development geographically.

Bibliometric analyses

Statistical analyses were conducted within the following fields: keywords, authors, corresponding authors, author countries or regions, journal names, and total citation frequency. A special evaluation was done to assess the annual MR training module development. The frequencies of the represented countries and the authors' affiliated countries were determined on the basis of the manually extracted data from each publication. The extent of collaboration among countries was manually extracted and augmented with a visual representation through VOSviewer. Similarly, the keywords of each article were visualized through a network map. Citation frequency stratified by year and country of publication was extracted and confirmed by searching the citation number of each publication through Google Scholar (Google LLC, California, United States). The author affiliation locations were also explored, and the numbers of single- or multiple-country publications were determined. The last analysis included the frequency of articles based on the publishing journal.

Results

The systematic literature review initially identified 1,063 articles from the three databases. After 127 duplicate results were removed, 936 articles were reviewed for relevance. Twenty-five articles were included in this systematic literature review; 17 (68%) involved cranial MR use, two (8%) described spine MR use, and six (24%) described both cranial and spine MR use or unspecified neurosurgical use (Table 1, Figure 1) [5-30].

**Table 1:** Summary of systematic literature review findings for 26 MR publications, January 1, 2013, to January 31, 2025 AR: augmented reality; CG: conventional group; MIMIT: mobile internet-based mixed-reality interactive telecollaboration; MR: mixed reality; NA: not applicable; VR: virtual reality Oculus Quest 2 (Meta Platforms, California, United States); iOS (Apple Inc., California, United States); ProDelphus (Pro Delphus Surgical Simulators, Olinda, Brazil); Microsoft HoloLens (Microsoft Corporation, Washington, United States); Magic Leap (Magic Leap, Inc., Florida, United States); Google Scholar (Google LLC, California, United States)

Subspecialty, authors, year	Institution location(s)	Study type	No. of participants	MR device(s)	Indication	Findings
Cranial						
Wang et al., 2024 [5]	China, Germany	Case series	40	HoloLens or iOS device	Tumor or neurovascular compression education	The MR neurosurgical training system developed by the authors may effectively help neurosurgeons in patient-specific training and planning of surgery for cases of neurovascular compression and intracranial tumors
Eom et al., 2024 [6]	United States	Case series	49 Participants with a patient-specific 3D-printed skull phantom	HoloLens 2	Extraventricular drain placement education	Real-time quantitative and visual feedback of an MR-guided craniostomy procedure can improve procedural accuracy and may be a valuable tool for trainee education and clinical implementation
Jean et al., 2024 [7]	United States	Case series	100	Not reported	Complex cranial cases; 60 mass lesions, 40 vascular indications	MR can aid by providing intraoperative guidance and tailoring operations based on patient-specific anatomy; no statistically significant differences exist between MR use and non-MR use regarding blood loss, length of stay, or duration of surgery
Cuba et al., 2024 [8]	Switzerland	Case series	6 Neurosurgical residents	Not reported	Intracranial aneurysm clipping	Training with an MR modality decreased clipping time and number of clipping attempts (p=0.02, p=0.03), and microscopic skills improved (p=0.03)
Silvero Isidre et al., 2023 [9]	Germany	Quasi-experimental	223 Medical students (120 in CG, 103 in MR group)	Magic Leap	Education	MR users were significantly more satisfied than CG users (p<0.001). These findings encourage the adoption of disruptive technologies into medical school curricula; MR may be an effective complementary tool to expose students to neurosurgery
Liang et al., 2023 [10]	Singapore	Proof of concept	30 (5 neurosurgeons, 8 neurosurgical trainees, 6 junior doctors, 11 medical students)	Oculus Quest 2 and HoloLens	Pediatric brain tumor education	The MR platform was a useful tool in various aspects of understanding pediatric brain tumors; further development to refine the current setup is needed
Colombo et al., 2023 [11]	Switzerland	Comparative study and questionnaire	107 patients (63 tumors, 27 cerebrovascular, and 17 carotid endarterectomy patients)	HoloLens 2	Tumor, cerebrovascular, and carotid endarterectomy	The perioperative use of 3D holograms improved direct anatomic visualization while not significantly increasing intraoperative surgical preparation time compared to a matched 2021 cohort
Peng et al., 2023 [12]	China	Proof of concept and comparative study	16	HoloLens	Neurosurgical ventricular and hematoma puncture training, education	Participants trained by this MR system were more familiar with localizing the lateral anterior ventricle horn puncture and the common endoscopic surgery for basal ganglia hemorrhage (p<0.05); these participants were more confident in the mastery of these operations compared with participants taught with traditional training methods only (p<0.05)
Raffa et al., 2023 [13]	Italy, Bulgaria, Romania, Sweden, Denmark, Austria, Israel, Portugal, United Kingdom, Russia, Serbia, Germany, Switzerland, Spain	Survey	441	NA	Education	Simulations based on AR/VR/MR were considered less valuable tools, being rated below sufficiency by 39.7% of responders
Yan et al., 2023 [14]	China	Case report	1	HoloLens 2	Intracerebral aneurysm clipping	MR systems can be used in microsurgical planning and education
Coelho et al., 2019 [15]	Brazil	Proof of concept	18 (14 pediatric neurosurgeons, 4 craniofacial plastic surgeons)	NA	Craniosynostosis repair education	More than 94% of participants found the MR simulator useful, considering weight, surgical positioning, dissection by planes, and cranial reconstruction; more than 60% of the surgeons approved the consistency and material resistance; MR systems can be used to achieve adequate psychomotor and cognitive skills
Jain et al., 2023 [16]	Singapore	Case series	3	HoloLens 2	Tumor	HoloLens 2 can easily overcome registration in the prone position compared to conventional neuronavigation; HoloLens 2 is a feasible device for intraoperative visualization of neurosurgical pathology
Zhang et al., 2022 [17]	China, Germany	Case series	20	MIMIT	Neuroendoscopy procedures education	The average video delay time was 184.25 msec (range, 160-230 msec) with 4G mobile internet and 23.25 msec (range, 20-26 msec) with 5G mobile internet; the MIMIT system allows for real-time, long-distance telecollaborative neuroendoscopic procedures and surgical training through a commercially available and inexpensive system
Zhou et al., 2022 [18]	China	Proof of concept and clinical trials	NA	HoloLens	Tumor	Accuracy of spatial registration was 1.18 mm in the phantom experiments and 1.86 mm in the 16 clinical trials; the experimental results indicate that this system has suitable accuracy and efficacy for clinical usage
Stifano et al., 2023 [19]	Italy, United States	Questionnaire	10	HoloLens	Intracranial unruptured aneurysm	Participant feedback showed MR platform advantages in educational value, craniotomy planning, and anatomical imaging interpretation during surgery; graphic performance was satisfactory; disadvantages were that the device was not as easy to use as expected and was uncomfortable when worn for a long time
Liu et al., 2021 [20]	China	Proof of concept	1	HoloLens	Brain metastasis brachytherapy	The 3D hologram offered good visualization of the skull, tumor location, and main vessels around the tumor; D90 and V100 of the postoperative plan and preoperative plan were 131.8 Gy vs. 132.0 Gy and 94.8% vs. 94.0%, respectively
Hooten et al., 2014 [21]	United States	Questionnaire	260 Residents	University of Florida Mixed Ventriculostomy Simulator	Education for ventriculostomy	University of Florida Mixed Ventriculostomy Simulator was used to assess the validation of this MR tool as a necessary training tool in neurosurgical residency; results showed that most residents agreed the simulator was realistic and that training with the simulator was beneficial and could increase patient safety; this institution now makes residents prove efficiency on the simulator before interacting with patients
Spinal						
Pose-Díez-de-la-Lastra et al., 2023 [22]	Spain, Canada	Proof of concept	6	HoloLens 2	Pedicle screw placement	The MR system had a mean error of 2.1 ± 1.4 mm; 98% of the screw placements performed with the MR system were successful
Goelho and Defino, 2018 [23]	Brazil	Questionnaire	16 Spine surgeons	Physical and virtual simulators produced by ProDelphus	Education	Most participants (94%) felt that the simulator provided a field for developing skills and altered the attitudes of trainee surgeons
Giraldo et al., 2025 [24]	United States, Colombia	Prototype and proof of concept	1	HoloLens	Education for spine freehand pedicle screw placement	A freehand pedicle screw MR placement prototype was used to validate pedicle screw bridges through standardized scoring systems and skill acquisition tracking
Both or unspecified						
Gupta et al., 2024 [25]	United States	Questionnaire	116 Residents and fellows	Not reported	Education	Agreement among neurosurgical trainees that extended reality (AR, VR, and MR) has potential as a training modality in neurosurgical education
Wach et al., 2024 [26]	Germany	Questionnaire	150 Medical students	HoloLens	Education	Students exhibited significant interest and willingness to engage in MR in neurosurgery; 94.7% of students agreed that MR may enhance their understanding of operative neuroanatomy
Ganeshkumar et al., 2024 [27]	India, Italy	Questionnaire	22	HoloLens 2	Craniovertebral junction education	MR is an effective teaching tool for craniovertebral junction pathoanatomy for young neurosurgical trainees
Jain et al., 2023 [28]	United Kingdom, Singapore	Questionnaire	8	HoloLens 2	Education	MR platform use in neurosurgery training is feasible without significant preparation requirements
Kos et al., 2023 [29]	The Netherlands, Switzerland	Questionnaire	15 Nonsurgical operating room staff	HoloLens 2	Various neurosurgical procedures	MR was considered an adjunct to improve operating room efficiency; further understanding of the impact of MR implementation for the nonsurgical staff could lead to targeted improvement of its use and potentially increase teamwork quality
Bova et al., 2013 [30]	United States	Prototype design and proof of concept	NA	University of Florida, 3 simulator developments	Simulation development	Simulators for ventriculostomy, percutaneous stereotactic lesion procedure for trigeminal neuralgia, and spinal instrumentation were created

The most common indication was education in 14 publications (nine cranial, one spinal, and four combined cranial and spinal, or unspecified neurosurgical uses). The Microsoft HoloLens was the most common MR device used in these studies (15), and education (13) was the most reported indication for its use or development.

The 25 articles analyzed included geographic locations and institutions with MR modality development in 21 countries, with the following frequencies in North America. The United States contributed six articles (13%) with 38 of 181 authors (21%), and Canada contributed one article (2%) with three authors (2%). In South America, Brazil contributed two articles (4%) with seven authors (4%). European contributions included Germany with five articles (11%), Switzerland with four (9%), Italy with three (7%), the United Kingdom with two (4%), Spain with two (4%), and Bulgaria, Romania, Sweden, Denmark, Austria, Portugal, Russia, Serbia, and the Netherlands with one article each (2%). China led Asia with six articles (13%) and 44 authors (24%), Singapore contributed three articles (7%), and India contributed one article (2%). Israel also contributed one article (2%).

One hundred eighty-one authors from 21 countries contributed to the 25 publications analyzed. One author was affiliated with both Sweden and Denmark. The highest number of authors had affiliations in China or the United States, with 44 (24%) total authors from China and 38 (21%) from the United States.

**Figure 2:**
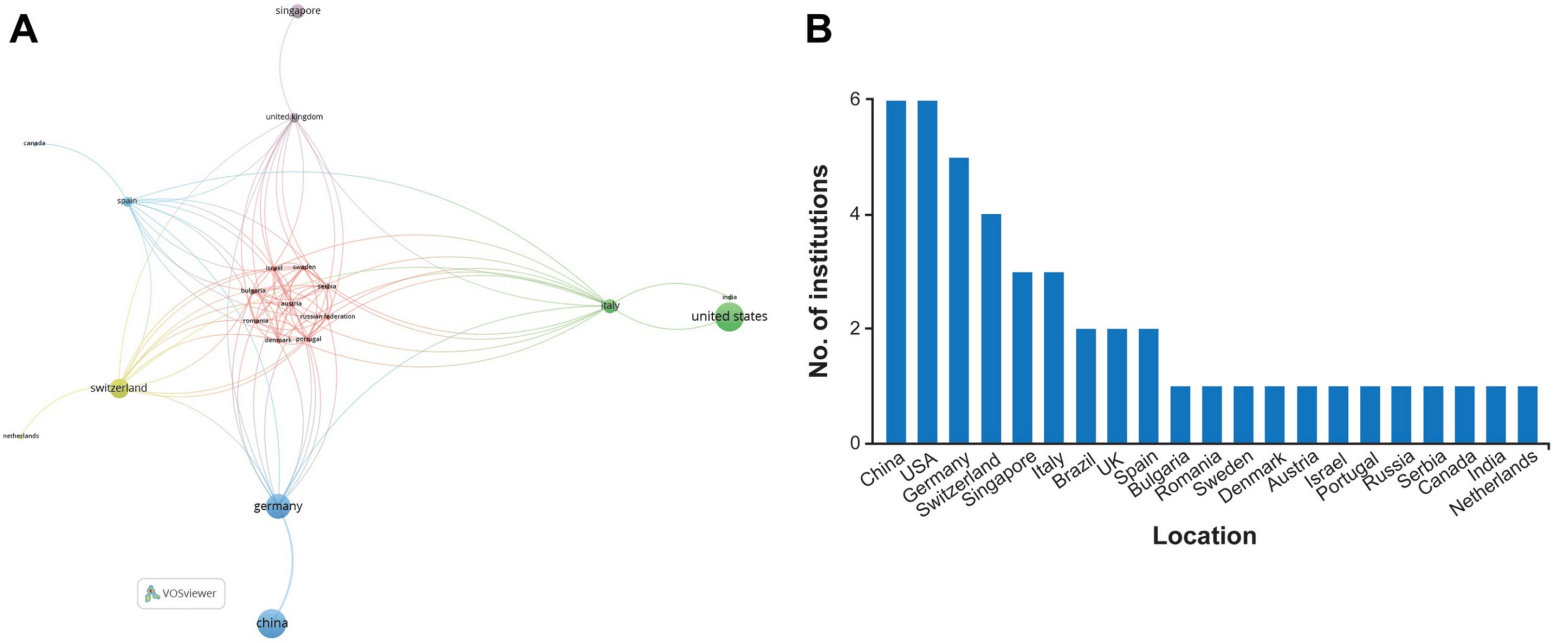
Network visualization map of global collaboration and journal data showing the number of publications on neurosurgical mixed reality (MR) A: network visualization map of global collaboration in neurosurgical MR development created by the VOSviewer tool (Centre for Science and Technology Studies, Leiden University, The Netherlands) [4], revealing the global collaboration between countries that have developed MR neurosurgical technology; B: journal data showing the number of publications on neurosurgical MR applications by country, January 1, 2013, to January 31, 2024. Used with permission from Barrow Neurological Institute, Phoenix, Arizona.

Global collaboration was observed among all countries except Brazil, with two independent publications on neurosurgical MR development. China (four), the United States (five), Switzerland (two), and Germany (two) also independently published on neurosurgical MR development and had other instances of global collaboration. Although the United States and China were the most represented in neurosurgical MR development, with six papers each, they had only one link to Italy and one to Germany, respectively. Although Italy had only three publications, it had 15 links to various countries, followed by Switzerland, Germany, Spain, and the United Kingdom, with 14 links each (Figure 2) [4].

Among corresponding authors, the countries with the highest number of publications in neurosurgical MR development also followed this publishing trend. The United States had four single-center publications based on the corresponding authors’ locations, and Singapore, Germany, Brazil, and Switzerland had two each. The one exception was China, which had four single-center publications based on the corresponding author and two multiple-center publications (Table 2).

**Table 2:** Global distribution of MR articles published from January 1, 2013, to January 31, 2024, by neurosurgical application, author, independent production, and global collaboration Data are no. (%) unless otherwise indicated. Percentages may not total 100% and the column totals may not correspond to the respective n value in each column because each article may have multiple country affiliations. Authors’ nationalities were determined by their hospital affiliations. When authors with various nationalities produced an article, nationality was attributed to each affiliated country. The number of links to other countries represents the number of international co-authorships identified using VOSviewer (Centre for Science and Technology Studies, Leiden University, The Netherlands). A link was established if at least one co-author from a different country was listed on a publication.

Country	Articles (n=25)	Cranial MR cases (n=33)	Spinal MR cases (n=3)	Cranial and spinal cases (n=9)	Total authors (n=181)	Independent publications (n=17)	No. of links to other countries
China	6 (24)	6 (18)	0	0	44 (24)	4 (23)	1 (Germany)
United States	6 (24)	4 (12)	0	2 (22)	38 (21)	5 (29)	1 (Italy)
Germany	5 (20)	4 (12)	0	1 (11)	15 (8)	2 (12)	14
Switzerland	4 (16)	3 (9)	0	1 (11)	25 (14)	2 (12)	14
Singapore	3 (12)	2 (6)	0	1 (11)	0	2 (12)	0
Italy	3 (12)	2 (6)	0	1 (11)	11 (6)	0	15
United Kingdom	2 (8)	1 (3)	0	1 (11)	4 (2)	0	14
Spain	2 (8)	1 (3)	1 (33)	0	3 (2)	0	14
Brazil	2 (8)	1 (3)	1 (33)	0	7 (4)	2 (12)	0
Bulgaria	1 (4)	1 (3)	0	0	11 (6)	0	0
Romania	1 (4)	1 (3)	0	0	1 (0.6)	0	0
Sweden	1 (4)	1 (3)	0	0	1 (0.6)	0	0
Denmark	1 (4)	1 (3)	0	0	1 (0.6)	0	0
Austria	1 (4)	1 (3)	0	0	1 (0.6)	0	0
Israel	1 (4)	1 (3)	0	0	1 (0.6)	0	0
Portugal	1 (4)	1 (3)	0	0	1 (0.6)	0	0
Russia	1 (4)	1 (3)	0	0	1 (0.6)	0	0
Serbia	1 (4)	1 (3)	0	0	1 (0.6)	0	0
Canada	1 (4)	0	1 (33)	0	3 (2)	0	0
India	1 (4)	0	0	1 (11)	10 (6)	0	0
The Netherlands	1 (4)	0	0	1 (11)	1 (0.6)	0	0

**Figure 3:**
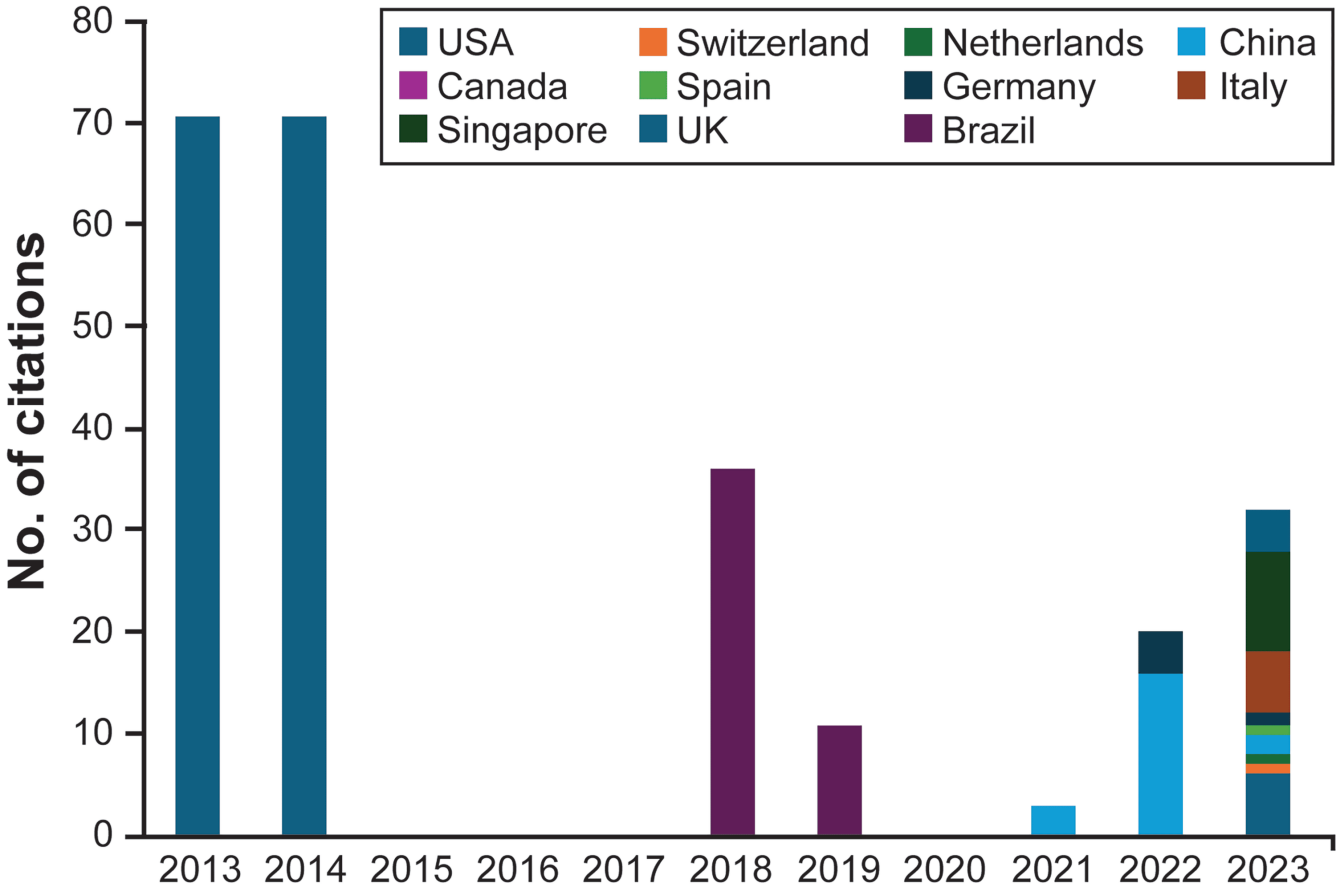
Citation numbers by year and country, January 1, 2013, to January 31, 2024 Used with permission from Barrow Neurological Institute, Phoenix, Arizona.

Citation frequency was the greatest in 2013 and 2014, with 71 citations each year of publications from the United States. Since then, there has been a downward citation trend, with isolated peaks of increased frequency in 2018, through a paper published in Brazil, and in 2023, among multiple countries (Figure 3).

Over this study period, the United States had the greatest number of citations (150), followed by Brazil (47), China (21), Singapore (10), Italy (six), Germany (five), the United Kingdom (four), and Switzerland, the Netherlands, Canada, and Spain (one citation each). The initial peak during 2013-2014 corresponds to foundational MR prototype studies, such as Bova et al. [30]. The 2018 and 2023 spikes align with broader accessibility of MR hardware and global investment in digital education, including the expansion of HoloLens-based platforms. The additional study from January 2025 revealed one additional connection between the United States and Colombia.

**Figure 4:**
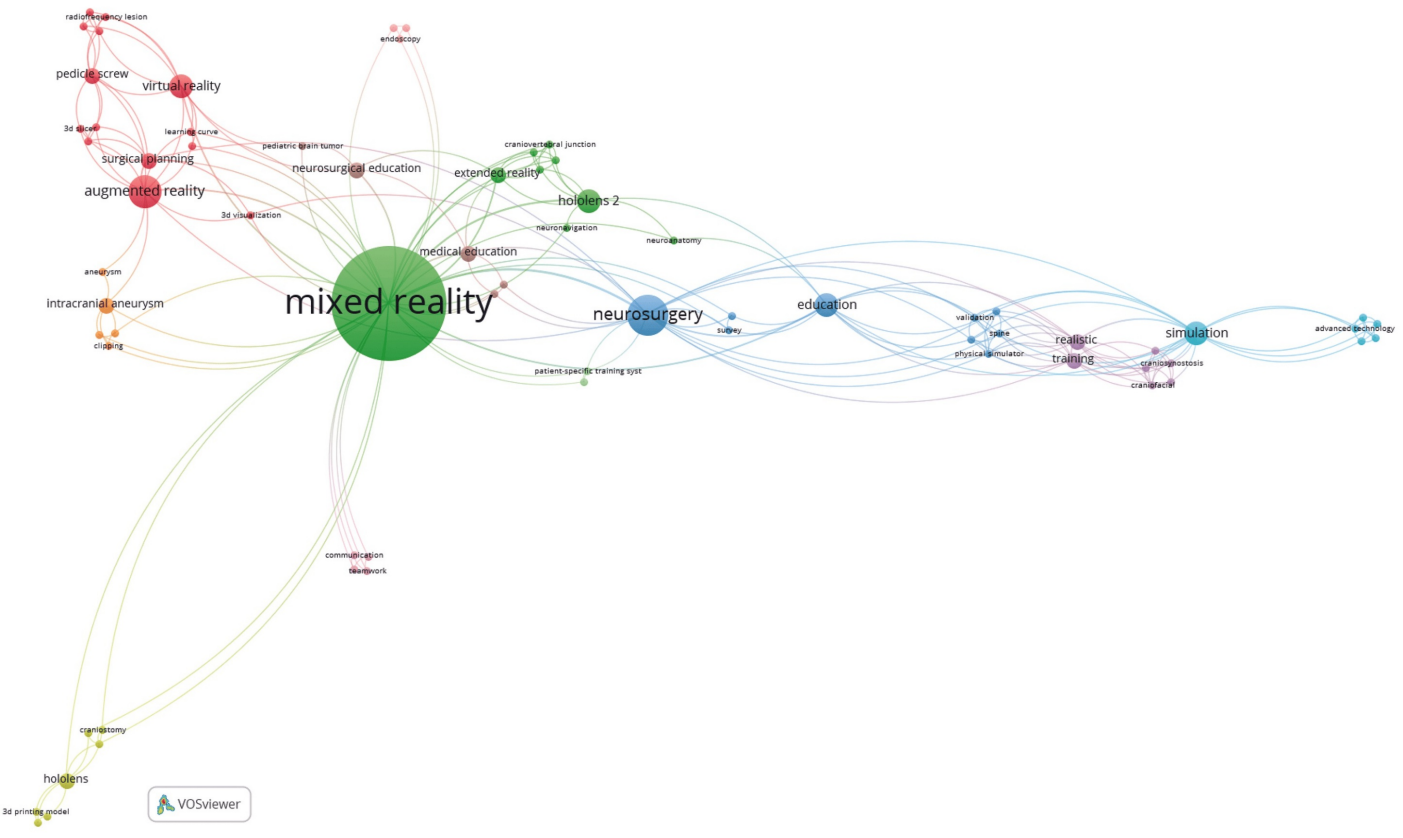
Network visualization map of keywords Constructed using the VOSviewer tool  (Centre for Science and Technology Studies, Leiden University, The Netherlands) [4], based on neurosurgical mixed reality publications from January 1, 2013, to January 31, 2024. Used with permission from Barrow Neurological Institute, Phoenix, Arizona.

Among the 25 publications, *Neurosurgical Focus* published six articles (24%), followed by *Operative Neurosurgery* with four (16%). *World Neurosurgery* contributed two publications (8%); the rest of the 13 were published in various journals (one each). Among these publications, the term “mixed reality” appeared most frequently in the keywords (14 times), with 40 links among other related keywords. Additionally, “neurosurgery” (used five times) and “augmented reality” (four times) were the second and third most common keywords, with 20 and 13 links, respectively (Figure 4) [4].

Over time, keyword usage transitioned from general terms like “simulation” or “augmented reality” toward more specific educational contexts such as “training,” “planning,” and “patient-specific models,” reflecting a maturing field with more targeted applications. The inclusion of the additional article, bringing the total to 26 studies for the 2025 update, did not alter the results in terms of citations or authorship. However, it revealed a new connection between Colombia and the United States. This study did not impact the overall bibliometric analysis because an increase in citations, author frequency, and collaborations was anticipated.

Discussion

The globalization of MR as a complementary modality of surgical and anatomical teaching strengthens global collaboration among neurosurgeons and trainees in the development and mastery of operative skills. As the use of MR in neurosurgery education and training expands and pushes the boundaries of neurosurgical training, worldwide collaborations grow [31]. International collaboration based on neurosurgical education has been shown to be effective in training physicians and can enhance neurosurgical training programs.

International global MR collaboration may also aid in sharing information across institutions from the surgeon’s perspective. The mobile internet-based mixed-reality interactive telecollaboration system developed by Zhang et al. may have a similar approach to allowing rapid information sharing [17]. Surgeons may share their perspectives with other surgeons or trainees internationally using an MR modality. Demonstration of surgically challenging procedures or rare conditions may be instrumental in sharing information through the headset display and spatial mapping technologies of MR. Clinically, MR platforms enhance preoperative planning and procedural safety through spatial rehearsal, which may reduce operative time and improve accuracy. Educationally, MR enables iterative, self-paced learning in low-risk environments, making it a powerful adjunct in competency-based surgical training [24].

The bibliometric analysis revealed that the countries most represented in neurosurgical MR development (the United States and China) collaborated only among themselves. Although Italy had only three publications, it had the most collaborations, with 15 countries (Figure 2). The United States and China had the most single-center publications when analyzing the publications by corresponding author. Although China had two multiple-center publications, the countries with the most representation of neurosurgical MR development tended to collaborate less with other countries. Despite being global leaders in MR development, the United States and China show minimal collaboration, which may be due to regulatory differences regarding data sharing, proprietary constraints in technological development, or geopolitical differences. Encouraging bi-national educational consortia may mitigate these barriers. The analysis shows that pursuing global collaborations on neurosurgical innovation is relevant, especially because the countries with more involvement and potentially more resources collaborate differently than countries with less overall involvement, as seen in our analysis. However, future trends in MR development will elucidate whether the socioeconomic impact addresses a different gap in MR application development. Although the authors’ nationalities were not gathered in this study, it is foreseeable that authors from multiple countries have developed these emerging technologies inside clusters within the United States, China, and Germany. By highlighting global trends and collaboration gaps, this study underscores the need for MR tool accessibility, particularly in low- and middle-income countries, where scalable, cost-effective MR solutions can play a transformative role in neurosurgical education and training. Collaborations with these nations would enhance global neurosurgical training.

When examining citation trends, we found isolated peaks of increased citation frequency within the previous 12 years. These peaks represent boosts in MR development within neurosurgery that correlated with the introduction of the Google Glass device (Google LLC, California, United States), which became available in 2014. More recently, although there has been a decrease in the number of citations since 2014, there is an increase in internationally diverse institutions contributing to MR development (Figure 3). The increase in MR research and development could be due to better availability of the technology and decreased prices, especially the affordability of headsets (a trend that was observed after mass production of different headsets, leading to market redistribution). This fledgling trend of diverse collaboration could continue in the upcoming years because of the need for innovative neurosurgical education and virtual hands-on training. Additionally, the increasing numbers of countries and institutions participating in neurosurgical MR development complement the diversity of journals that have published these 25 studies and may help foster worldwide collaboration. Similarly, our analysis of keywords pinpointed the specific terminology used to find relevant publications in neurosurgical MR development (Figure 4). New neurosurgeons may benefit by understanding the most connected and prevalent keywords to gain better access to learning and appreciating this novel innovation in neurosurgery education and training. Our findings affirm the hypothesis that MR development is concentrated in resource-rich nations, with minimal contributions from low- and middle-income countries. The limited international collaboration further underscores the need for inclusive global neurosurgical education frameworks.

## Conclusions

MR is becoming an integral tool in neurosurgical education, with increasing global interest and institutional support. While research and development are concentrated in a few leading countries, international collaboration remains limited. Expanding these partnerships could enhance the accessibility and implementation of MR in neurosurgical training worldwide. Strengthening global cooperation will be essential for maximizing the impact of MR on surgical education and practice. Greater integration of MR into standardized curricula could refine surgical skills, improve trainee competency, and bridge educational disparities across institutions. As MR technology advances, fostering interdisciplinary collaboration and resource-sharing will be critical to optimizing its role in neurosurgical training and patient care.
